# Rations and Health

**Published:** 1918-02-02

**Authors:** 


### SOME PROBLEMS OF TO-DAY

### Rations and Health.

By A SOCIAL WORKER.

Voluntary rationing is established, and compulsion has to some extent begun at local centres. In some places, as at Birmingham, the plan appears to be having a wholesome effect upon queues, and therefore indirectly upon national health. Serious results must certainly follow a continuance or increase of the practice of putting children to keep the mother's place for an hour or two in these motionless processions during wintry weather. But whatever may be the result of rationing, whether voluntary or by local or national compulsion, the fact remains that a less amount of food all round per head needs to be used, and that some common articles of diet will be quite or almost out of reach for an increasing number of people.

### Combating Prejudice.

The question is, therefore, how to make the available most efficient, and especially for children, adolescents at work, grown people working hard, and delicate people who should, if possible, be prevented from becoming useless and overweighting the work of doctors. Friends of the poor and workers among them must especially ask what advice and help they can give in these matters. Lurid stories of prejudice against wholesome but unaccustomed foods are, of course, plentiful, and are sufficiently true to show the need for wise action, and those who have penetrated at least a little way into the workings of the uneducated mind will realise that a very severe pinch may be endured with dire results to the family before a working woman can be converted to a new idea. A few years ago the wife of a sick man who had greatly enjoyed a tasty dish of macaroni was invited to the donor's house to learn to prepare it for herself, but departed in wrath on finding that her poor husband had been given "that nasty foreign stuff" artfully disguised. Still older is the story of the London boy who refused country milk because instead of coming from a nice clean shop it was dragged out of a horrid cow. The tired waves will seem for some time to break very vainly on rocks like these, but at the present moment the full tide of dire necessity is flowing in behind them and there is hope.

### Exploit the Less-used Third.

The elaborate investigations made under high authority into food values have their uses, though "calories" are fearsome wild-fowl to everyone, probably, except a chemist. The conclusions drawn by the investigation carried out under the Ministry of Munitions into the food of workers wisely remind us that the rations set are an average for the whole nation, and therefore something of the surplus not needed by the infant or the aged may help those who are working and growing; it would, above all, be suicidal to underfeed children and make the next generation grow up weakly, under-developed, and prone to disease. At the same time it is now estimated that before the war bread, meat, and sugar made up two-thirds of the nation's food. Some expansion of the less-used third is evidently possible, such as the much-insisted-on potato, other vegetables, *raw* apples, cheese used hot and in combination with other things, and so forth. It is here especially that better cookery is needed. For instance, the experience of the Navy and Army Canteen Board has shown the ready enjoyment by men of many kinds of vegetable-and-cheese dishes, while such a garden product as beetroot doubles its value if given to children hot, instead of reduced to cold, peppery, and vinegary slices. Said a sensible working woman lately: "I tries all sorts of new ideas, and if they don't like 'em I waits a week and gives it to 'em again." On the value of suet pudding she was enthusiastic, varying it with treacle or gravy or jam or a few precious raisins, or rolling it round apples. *O si sic omnes!*

### Table Habits and Digestion.

For some difficulties there may, indeed, be a simpler remedy. The elect discover that if you must stand in a queue to get tea and margarine you can have coffee and dripping in the ordinary way of commerce. These also make the wise use of communal kitchens by buying the *pièce de résistance* there, strong soup (which may even need to be thinned for the younger children), solid pies and puddings, and rice, of which the nourishment has been doubled by proper—that is, long and slow and fuel-devouring—cookery. They can make use of fresh herrings and of sprats. They can be persuaded to steam a cabbage instead of boiling it to death. Above all, they know the value of set meals eaten with clean hands and faces and decent table manners, without haste, and with a careful avoidance of even necessary fault-finding or other moral east-wind influences. It is to spread these habits that is above all necessary, and is likely to lead in the adolescents to wise choices of food, less tea-and-bun lunches for the girls, less drinks and snacks for the lads, resulting in a better digestion through life, and that influence of good habits to which we are apt to attribute too little importance, while readily recognising the power of bad ones.* A few days ago two pretty little girls were munching large lumps of bread, roughly daubed with treacle, early in the afternoon. Their faces and hands were smeared from the street, the use of pocket-handkerchiefs was pressing, pieces fell from the food as they walked about. Oh, yes, they had dinner not long ago, the mother explained; they just asked for "a piece."

At this moment the idea of continued education for young girls employed in public offices is assuming practical shape in the daily hour of "lessons," which is a condition of employment for the "sugar-card girls." Is it too much to hope that clear teaching in practical domestic economy will come to be looked upon as an essential part of every girl's training, not less essential, let us say, than a knowledge of the alphabet or the multiplication table, much more so than the use of a typewriter? Nothing less than the national health is involved.

---

* "The new recruits," writes Dr. Leonard Hill of munition workers, "are anxious to create a good impression, and overtax themselves while they lunch on buns and cakes. When the worker learns to ask for and consume a good canteen plateful of meat and vegetables improvements in health and output will not be long in making their appearance."

